# Jaws of a large belemnite and an ammonite from the Aalenian (Middle Jurassic) of Switzerland

**DOI:** 10.1186/s13358-020-00207-7

**Published:** 2020-08-28

**Authors:** Christian Klug, Walter Etter, René Hoffmann, Dirk Fuchs, Kenneth De Baets

**Affiliations:** 1 Paläontologisches Institut und Museum, Universität Zürich, Karl-Schmid-Strasse 4, Zurich, 8006 Switzerland; 2 Naturhistorisches Museum Basel, Augustinergasse 2, Basel, 4001 Switzerland; 3 Institute of Geology, Mineralogy & Geophysics, Ruhr-Universität Bochum, Bochum, 44801 Germany; 4 SNSB-Bayerische Staatssammlung für Paläontologie und Geologie, Richard-Wagner-Straße. 10, Munich, 80333 Germany; 5 GeoZentrum Nordbayern, Fachgruppe PaläoUmwelt, Universität Erlangen, Loewenichstr. 28, Erlangen, 91054 Germany

**Keywords:** Cephalopoda, Megateuthididae, Graphoceratidae, Mouthparts, Body size, Opalinus clay

## Abstract

Although belemnite rostra can be quite abundant in Jurassic and Cretaceous strata, the record of belemnite jaws was limited to a few specimens from Germany and Russia. Here, we describe and figure three cephalopod jaws from the Middle Jurassic Opalinus Clay of northern Switzerland. Although flattened, the carbonaceous fossils display enough morphological information to rule out an ammonoid, nautiloid or octobrachian origin of the two larger jaws. Their similarities to belemnite jaws from Germany and Russia conforms with our interpretation of these specimens as belemnite jaws. Based on their rather large size, we tentatively assign these two jaws to the megateuthidid *Acrocoelites conoideus*. The third jaw is a rather small upper jaw of an ammonoid. Since *Leioceras opalinum* is by far the most common ammonite in this unit in northern Switzerland, we tentatively suggest that the upper jaw belongs to this species.

## Introduction

Belemnites are extinct coleoid cephalopods, i.e., relatives of modern squids, cuttlefishes and octopuses (Fuchs 2006; Kröger et al. 2011; Iba et al. 2012, 2014; Klug et al. 2016; Hoffmann et al. 2016, 2020; Hoffmann and Stevens 2020). With the ten-armed coleoids, the decabrachians, they share an internal skeleton largely surrounded by a muscular mantle, a large brain compared to other invertebrates, ten arms, chitinous jaws, large lateral eyes, and a predatory mode of life (e.g., Naef 1922; Reitner and Urlichs 1983; Doguzhaeva et al. 2002, 2003; Weis and Delsate 2006; Klug and Fuchs 2010; Klug et al. 2010a, b, 2016; Keupp and Mitta 2015; Clements et al. 2016; Donovan & Fuchs 2016; Klug and Tajika 2018; Wani et al. 2018; Jenny et al. 2019; Hoffmann and Stevens 2020). In contrast to other hard parts, the low magnesium calcite rostra of belemnites represent abundant fossils in the Jurassic and Cretaceous, sometimes occurring in rock-forming numbers (Doyle and Macdonald 1993; Rita et al. 2018). All other body parts are much less commonly preserved and true belemnite soft parts were not described before 1983 (Reitner and Urlichs 1983), after a series of fakes had been erroneously published as first records of belemnite soft parts (Huxley 1864; Wiesenauer 1976; Rietschel 1977; Seilacher and Wiesenauer 1978). Fossilized body parts include, in descending order according to the abundance of their preservation, remains of the phragmocone, arm hooks, remains of the mantle musculature, ink sac, proostracum and jaws. Jaws remains were first recognized by Reitner and Urlichs (1983), but their specimens only showed black patches near the bases of the arms, which lack morphological detail.

The first belemnite jaws revealing some more morphological information come from the Kimmeridgian of Nusplingen, Germany (for stratigraphic and locality details, see Klug et al. 2010b). This specimen (SMNS 67335) was assigned to *Hibolithes*, the most common species in these strata. In addition to the upper and lower jaw, it preserves the fractured phragmocone, a patch with ink, and a patch with many arm hooks, some of which are still aligned with the respective arm. The fractured phragmocone lacking the apex, the missing rostrum and the leaked ink suggest that the individual was a victim of failed but still lethal predation. Although the presence of chitinous jaws in belemnites is corroborated by poorly preserved remains from the Toarcian (Reitner and Urlichs 1983) and by their phylogenetic context (e.g., Klug et al. 2017), only very few remains of the buccal hard parts of belemnites were reported. Dzik (1986) described coleoid jaws from the Polish Callovian. These two jaws indeed resemble the jaws described here and thus are probably also belemnite jaws. Similarly, Keupp and Mitta (2015) published upper and lower jaws of Callovian belemnites, which are quite well-preserved in three dimensions. No further records of belemnite jaws are known to us.

Here, we present cephalopod jaws from the latest Toarcian (Early Jurassic) to Aalenian (Middle Jurassic) Opalinus Clay Formation of northern Switzerland. In southern Germany and northern Switzerland, the Opalinus Clay is exposed in several clay pits and a few natural exposures. This formation is characterized by a rather low benthic diversity, well-preserved arthropods and the rare preservation of articulated echinoderms and vertebrate skeletons (e.g., Etter 1988, 1990, 1995, 1996, 2004a, b). The name of this lithostratigraphic unit derives from the locally abundant ammonite *Leioceras opalinum*, whose species name refers to the opal-like nacre preservation widely found in central Europe (Quenstedt 1843). However, in northern Switzerland, the aragonite is always dissolved whereas the organic periostracum of the ammonites and other molluscs is preserved. Compaction of the sediment was strong and the ammonites are usually completely flattened. The preservational patterns of the Opalinus Clay fossils are thus similar to those of the Toarcian Posidonia Shale. Belemnite rostra are fairly common (Etter 1990). The jaws portrayed here were recognized as coleoid jaws by Etter (1990) but never documented in detail. Accordingly, the aims of this paper are to (1) document the isolated cephalopod jaws from the Swiss Opalinus Clay Formation, (2) to compare them to the few other records of Jurassic coleoid jaws and (3) to assign them to Middle Jurassic cephalopod taxa as far as possible.

## Material

**Fig. 1:**
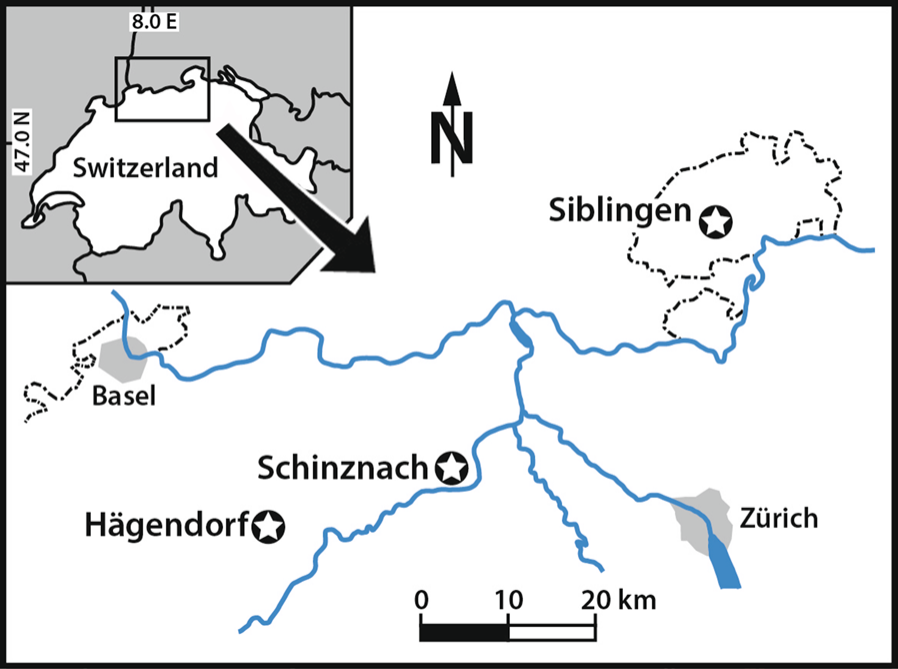
Location of the sampling sites in the Opalinum Clay Formation of northern Switzerland. The stars indicate the sampling sites. The dotted line is the Swiss border, which partially continues in the Rhine (see small map)

All cephalopod jaws and rostra are stored in the Paläontologisches Institut und Museum of the University of Zurich (PIMUZ number). They were collected by WE in 1985 at Eriwies near Schinznach in the Swiss canton Argovia (Fig. 1). All three jaws are from the lower part of the Opalinus Clay Formation, which is early Aalenian in age, *Leioceras opalinum* Subzone (earliest Middle Jurassic; further to the west and northwest, deposition of the Opalinus Clay Formation already started in the late Toarcian; Reisdorf et al. 2014; Hostettler et al. 2017). Various rostra were also collected by WE between 1985 and 1987 during field work for his dissertation at the localities Eriwies near Schinznach (canton Aargau), Fasiswald near Hägendorf (canton Solothurn) and Tenterenberg near Siblingen (canton Schaffhausen; Fig. 1). Of these, we illustrate rostra of the two common taxa *Acrocoelites quenstedti* (Oppel 1858) and *Acrocoelites conoideus* (Oppel 1857).

### Geological setting and taphonomy

The Opalinus Clay of southern Germany and northern Switzerland is of economic interest because it is in great parts a rather pure clay. It is used as additive in cement production, and for the production of bricks and foamed clay. Currently, it is under consideration for deep storage of radioactive waste in Switzerland (Marschall and Giger 2014).

In northern Switzerland, these dark grey claystones are well bedded and form a thick succession of around 100 m (Allia 1996; Wetzel and Allia 2003; Marschall and Giger 2014). The clay may contain trace fossils, current-induced sedimentary marks, and pyrite. Some layers contain various amounts of mica, silt, quartz sand and carbonate as well as concretions of varying composition (e.g., Allia 1996; Wetzel and Allia 2003; Reisdorf et al. 2014; Hostettler et al. 2017). The silt and sand content increases towards the top of the formation. Accordingly, three different types of sedimentary facies from bottom to top were recorded in Fasiswald by Reisdorf et al. (2014):

1. A shaly facies composed of pure clay, silty clay or clayey marls;
2. A sandy and carbonatic facies containing silty limestones and marls;
3. A sandy facies dominated by marly silts, silty marls and silty clays with siderite and bioclastic carbonate concretions.

Serious oxygen-depletion of the bottom-water and high sediment accumulation rates were responsible for the sometimes exceptional preservation of fossils including articulated arthropods, articulated echinoderms and vertebrates and preservation of organic materials like the ligament of bivalves and the periostracum of ammonites as well as other molluscs (e.g., Etter 1988, 1990, 1995, 2004a, b).

## Description

### Jaws

Here, we employ the terminology used by, e.g., Clarke (1962, 1986), Clarke and Maddock (1988), Klug et al. (2010b), Nixon (2015) and Tanabe et al. (2017) for the description of our jaw material.

**Fig. 2:**
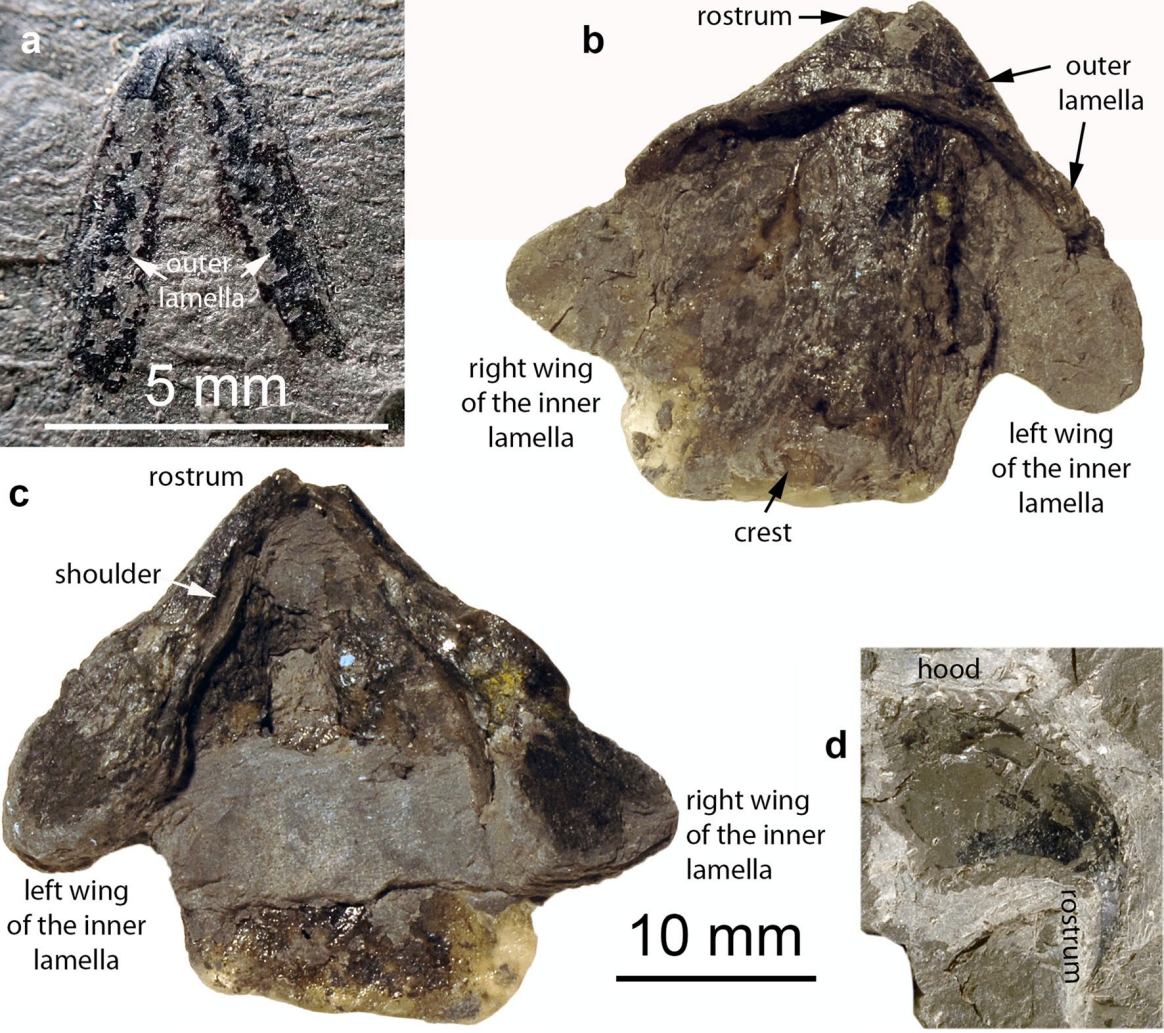
Cephalopod jaws from the Opalinum Clay Formation of northern Switzerland. **a** PIMUZ 5942, upper jaw of an ammonoid (*Leioceras opalinum*?). **b**, **c** PIMUZ 6077, lower jaw of a megateuthid belemnite (*Acrocoelites conoideus*?); **b** ventral view, **c** dorsal view. **d** PIMUZ 6076, upper jaw of a megateuthid belemnite (*M. rhenana*?), seen laterally

Upper jaw, PIMUZ 6076 (Fig. 2d): this specimen is largely laterally flattened, retaining only a little bit of its lateral three-dimensionality. The fact that it was embedded laterally represents a taphonomic support of its interpretation as an upper jaw, because most upper jaws are narrower than the corresponding lower jaws. Its greater height-to-width ratio makes it more likely to come to a rest on its side in contrast to the proportionally broader lower jaws. This is logical since the upper jaw is almost completely surrounded by the lower jaw in all cephalopods (in contrast to parrots, where it is the opposite). The largest extension of this jaw is 18 mm. Its nearly straight posterior edge is about 10 mm wide. The oral tip of the jaw, the so-called rostrum (not to be confused with the calcitic belemnitid rostrum), is hook-like, very narrow and elongate, downward bent and covered in carbonaceous material (previously chitinous). This cover becomes thinner posteriorly and dorsally. The dorsal posterior half of the jaw has only small patches remaining of the original carbonaceous sheet. Along the dorsal edge of the hood, many longitudinal and transversal fractures are visible. The limit of the outer lamella is not discernible. The posterior half of the jaw displays very fine concentric growth lines, which run approximately parallel to the posterior edge of the hood as far as it is preserved. Since no offset between hood and inner lamella is discernible, we suggest that the inner lamella is not preserved or covered by the large hood.

Lower jaw, PIMUZ 6077 (Fig. 2b, c): this lower jaw also underwent compaction, which reduced its height (now ca. 3 mm) to an estimated 10 to 20% (based on its original height—around 20 mm—and comparison to other lower jaws of coleoids; the deformation is also evident from compacted ammonite phragmocones) of its original height. Nevertheless, it still retains parts of its three-dimensional form, facilitating the interpretation of its morphology. It is 33 mm wide and 28 mm long. Since the fossil is very fragile and parts had broken off at the posterior end, the cast was filled with a small amount of transparent resin, which is visible in Fig. 2b on the left (right lateral wall of the inner lamella) and at the posterior end of the inner lamella (in Fig. 2c at the bottom, posterior of the remaining clay). In this case, the outer and inner lamellae are well discernible. In ventral view (Fig. 2b), the external lamella is short and curves backwards laterally. In dorsal view, the biting surface is visible with a moderately sized rostrum. The rostrum is slightly turned upward. Behind the rostrum, the 1 to 2 mm wide shoulders of the outer lamella are well developed over about 12 mm on both wings. Posteriorly, the transition between inner and outer lamella forms broad rounded surfaces, reaching 6 mm in width. Much of the fossil retains a thin carbonaceous coating. Most of the surface displays fine wrinkles, which likely formed during compaction.

Upper jaw, PIMUZ 5942 (Fig. 2a): this specimen is strongly flattened and differs in several respects from the two described above. Accordingly, it probably belonged to a different cephalopod group. It is about 4.5 mm long and 4 mm wide. In spite of its dorsoventral distortion, it is still quite symmetrical, displaying both lateral walls of the inner lamella. The anterior edge is rounded. Where the two lateral walls meet, a narrow triangular field is visible, which is delimited by a low ridge. The entire jaw is covered by a thin carbonaceous layer, which appears the darkest in the anteriormost 0.5 mm. This part is also set off by a faint transverse line, likely marking the posterior limit of the inner lamella.

### Rostra (guard-like envelope of the posterior phragmocone)

The following species of belemnites from the Opalinum Clay Formation of northern Switzerland were listed by Etter (1990), which are revised to conform to Schlegelmilch (1998): *Megateuthis beneckei* Schwegler 1938, *Acrocoelites (Acrocoelites) quenstedti* (Oppel 1856), *A. (A.) conoideus* (Oppel 1858), *Arcobelus meta* (Blainville and Ducrotay de 1827), *Salpingoteuthis trisulcata* (Blainville 1827), *Brevibelus breviformis* (Voltz 1830), *Neoclavibelus subclavatus* (Voltz 1830), *N. neumarktensis* (Oppel 1858), *Rhabdobelus exilis* (d'Orbigny 1842). Among those, *Acrocoelites quenstedti* (Oppel 1858) and *Ac. conoideus* (Oppel 1856) are among the most common species (the material of *Ac. conoideus* was formerly misidentified as ‘*Mesoteuthis*’ *rhenana*, a species now included in *Megateuthis*). We accordingly presume that some of the cephalopod jaws described here belonged to one of those species (see discussion).

**Fig. 3:**
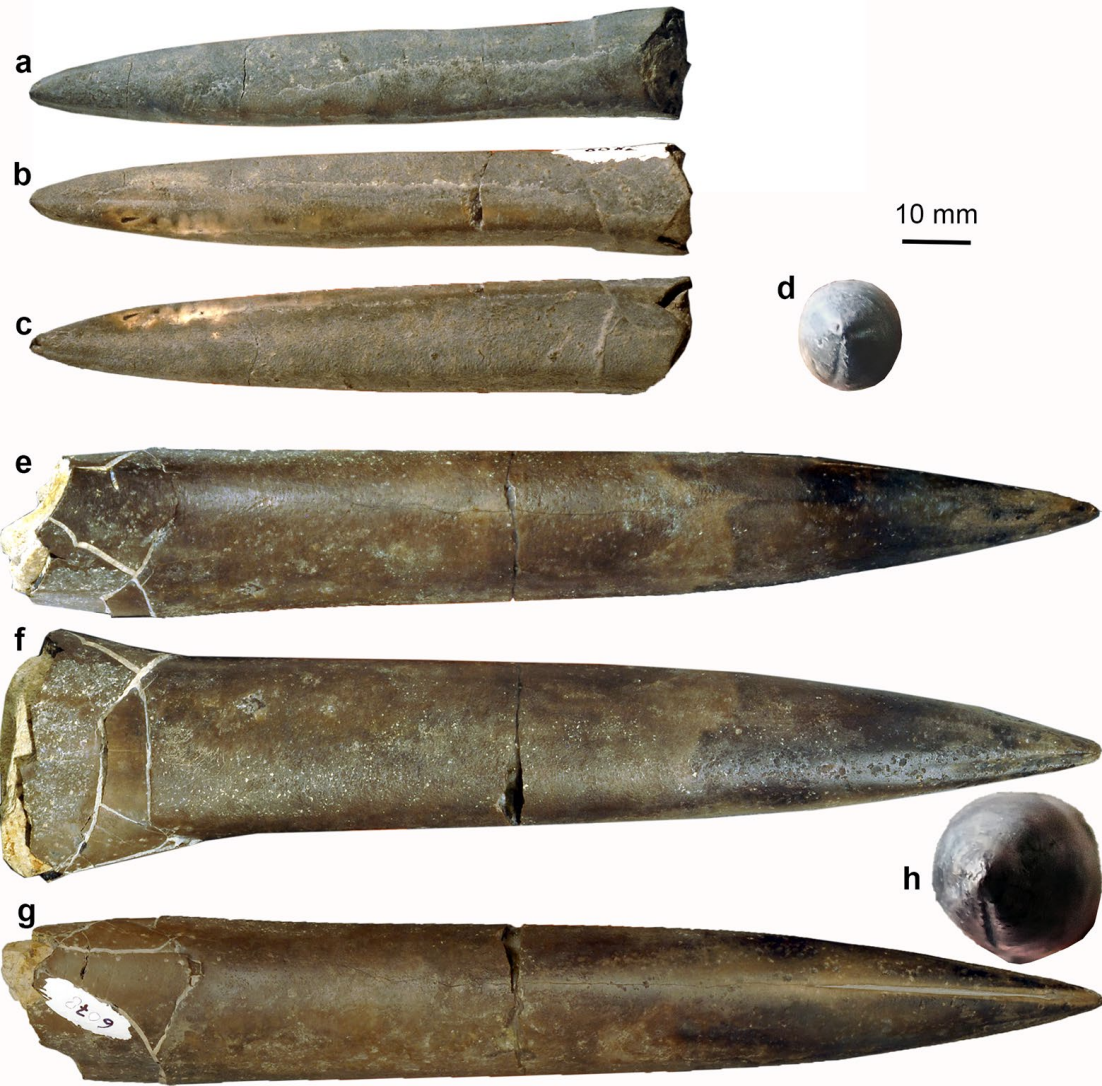
The most common belemnite rostra (Megateuthidae, Belemnitina) from the Opalinum Clay Formation of northern Switzerland. **a– d** *Acrocoelites quenstedti* (Oppel 1856), PIMUZ 6082. **a** dorsal view. **b** ventral view. **c** lateral view. **d** apical view. **e–h** *Acrocoelites conoideus* (Oppel 1856), PIMUZ 6078; large specimen. **e** dorsal view. **f** lateral view. **g** ventral view. **h** apical view

Rostrum of *Acrocoelites quenstedti* (Oppel, 1856), PIMUZ 6082 (Fig. 3a–c): This specimen is 95 mm long, 11 mm wide (in the alveolar region reduced to 10 mm) and maximally 12 mm high. It is undeformed, only the rostrum cavum is slightly fractured. The rostrum is cylindrical with a slightly compressed cross section and an acute to slightly rounded apex. Its profile is almost perfectly symmetrical. The apex shows a distinct ventral groove (38 mm long) and shorter paired dorsolateral grooves (ca. 15 mm long).

Rostrum of *Acrocoelites conoideus* (Oppel 1856), PIMUZ 6078 (Fig. 3d–f): This specimen is the largest available to us from the Swiss Opalinum Clay (cf. Rita et al. 2019). It measures 169 mm in length, 23 mm in width (barely constricted in the alveolar region) and maximally 23 mm in height. It is undeformed, only the rostrum cavum is fractured with the shards more or less in place. The rostrum is cylindrical with a barely compressed cross section and an acute apex. Its profile is nearly symmetrical. The apex shows a distinct ventral groove (45 mm long, fading out over another 16 mm), shorter, less sharp, paired ventrolateral grooves (ca. 30 mm long), even shorter dorsolateral grooves (19 mm long), which are slightly asymmetrical, and a faint dorsal groove.

## Discussion

Fossil coleoid jaws are poorly known globally. This is mainly due to their chitinous composition, which results in a significantly lower fossilization potential compared to mineralized carbonaceous hardparts (Nixon 2015; Donovan and Fuchs 2016). Although remains of belemnite mandibles were first recognized as such by Reitner & Urlichs (1983), the first remains of belemnoid jaws revealing morphological details were recorded much later by Klug et al. (2010b) and by Keupp and Mitta (2015). Overall, fossilized ammonoid jaws are quite well-known from Mesozoic Ammonoidea (e.g., Lehmann 1972; Kruta et al. 2011; Tanabe et al. 2015) and Nautilida sensu strictu (Saunders et al. 1978; Klug 2001), partially because many representatives have calcitic portions in their jaws. By contrast, jaws of Palaeozoic nautiloids (Turek 1978; Gabbott 1999), Palaeozoic ammonoids (e.g., Matern 1931; Closs 1967; Mapes 1987; Doguzhaeva 1999; Landman et al. 2010; Tanabe et al. 2015; Klug et al. 2016, 2017) and coleoids in general (Klug et al. 2005, Klug et al. 2010a, b, 2016, 2019) are still poorly known from only a low number of taxa. Moreover, from many Palaeozoic taxa like actinocerids, bactritids, endocerids, oncocerids, etc., jaws are still entirely unknown.

**Fig. 4:**
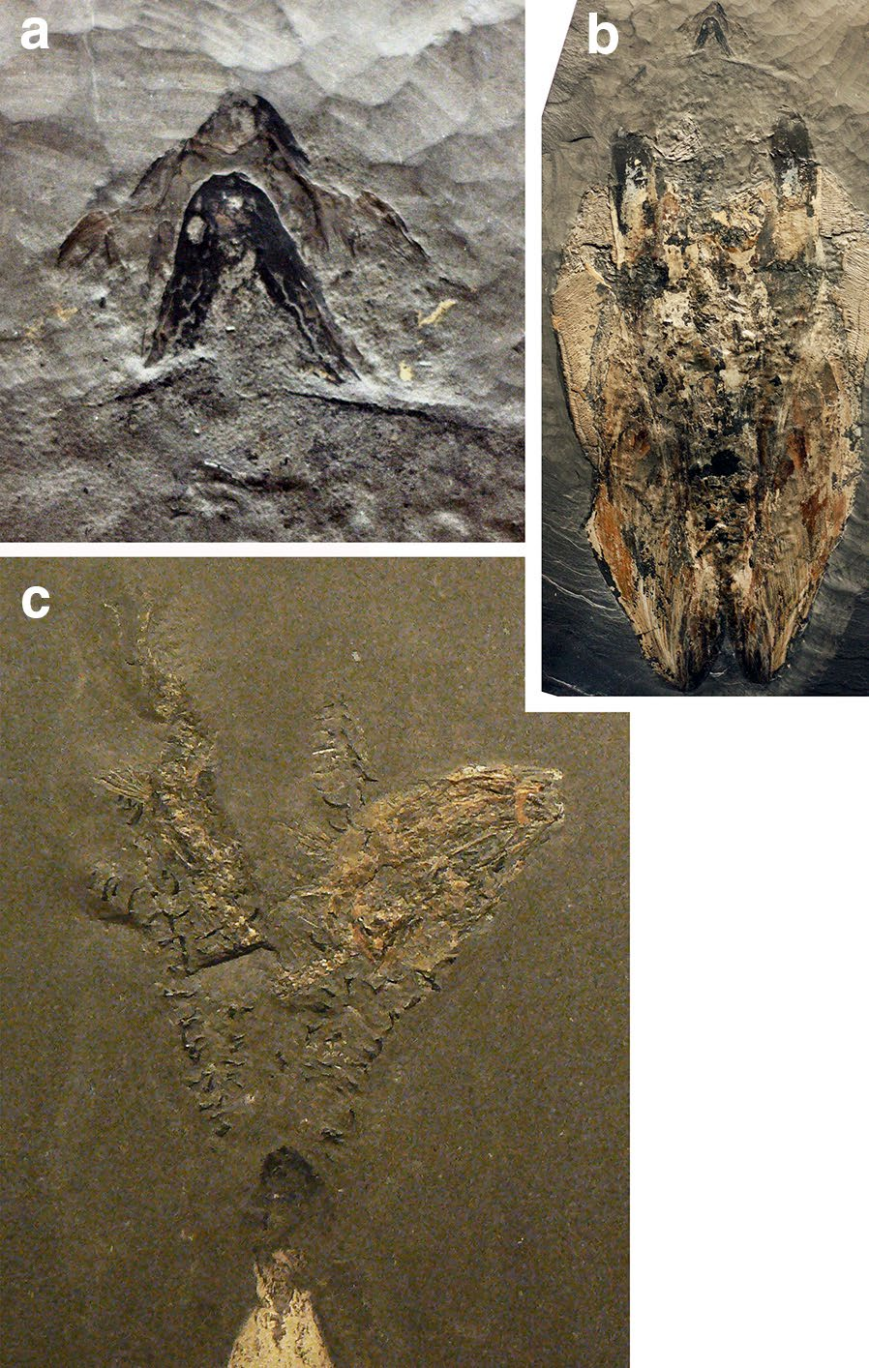
Coleoids from the Toarcian Posidonia Slate of Holzmaden; all specimens are on display in Holzmaden at the museum Hauff. **a**, **b** *Geopeltis simplex* (Voltz 1840), Loligosepiina, Octobrachia, gladius length ca. 200 mm. **a** detail of **b** showing the articulated jaws (ca. 20 mm long). **b** complete specimen with jaws and gladius. **c** *Clarkeiteuthis conocauda* (Quenstedt 1843), Diplobelida, detail from Jenny et al. (2019) showing its prey and the poorly preserved jaws; height of image ca. 100 mm

Accordingly, new discoveries of, in this case, coleoid jaws represent a welcome addition to our patchy knowledge. In the long lateral lappets, the small triangular field in the middle and the deep posterior incision in the inner lamella, the upper jaw PIMUZ 5942 (Fig. 2a) resembles the upper jaw of Carboniferous *Glaphyrites* illustrated by Bandel (1988) and even more so the upper jaws of, e.g., Cretaceous *Placenticeras* shown by Landman et al. (2006). Trauth (1930), Etter (1990) and Mitta et al. (2018) reported lower jaws of *Leioceras opalinum*. Mitta et al. (2018) illustrated isolated halves and they reconstructed the lower jaw (Fig. 6b). However, the upper jaw was not documented yet. Because PIMUZ 5942 is an upper jaw, it is quite elongate with a deep posterior incision allowing to move around the preceding whorl in the body chamber. Since *Leioceras opalinum* is by far the most common species (subordinately, lytoceratids occur) accounting with micro- and macroconchs for well over 95% of the ammonite specimens, we suggest that PIMUZ 5942 represents the upper jaw of this index ammonoid. There is some similarity to the upper jaw of *Geopeltis* (Fig. 4a), which is, however, unknown from the Swiss Opalinus Clay. Also, the jaw of the Toarcian coleoid is much larger and more rounded, while the upper jaw from the Opalinus Clay does resemble some ammonoid upper jaws.

For *Placenticeras*, Landman et al. (2006) suggested that this ammonite was able to use its jaws to grasp and cut small prey, as it was suggested for *Didymoceras* Kruta et al. (2010). Accordingly, *Leioceras opalinum* might have lived on a similar diet of small prey animals (microphagous).

The lower jaw PIMUZ 6077 (Fig. 2b, c) differs from jaws of Jurassic ammonoids in its broad and overall triangular shape with its small upturned rostrum. It cannot be a nautilid mandible because it lacks the calcitic conchorhynch (which should be preserved in these strata) and the characteristic striped occlusal surface. Gladius-bearing octobrachians (vampyromorphs and octopodids) are still unknown from Aalenian deposits, likely due to their low preservation potential (e.g., Clements et al. 2016). Owing to some similarities and the coleoid relationship, octobrachian affinities must be considered, because these coleoids were widely distributed during the European Mesozoic. The lower jaw morphology of PIMUZ 6077 is similar to the gladius-bearing vampyromorph *Trachyteuthis* in the posteriorly widened wings of the outer lamellae (Klug et al. 2005), but differs in the large inner lamella and the distinct rostrum. Upper jaws of vampyromorphs such as *Trachyteuthis* and *Plesioteuthis* (Klug et al. 2005, 2015) are similar in morphology to that of belemnitids in arrangement and overall proportions of the outer and inner lamella to belemnitids (Klug et al. 2010b), but belemnitids have a proportionally longer hood and a ventrally elongated rostrum (Klug et al. 2010b; Keupp and Mitta 2015). Accordingly, it shares the broad lateral walls of the inner lamella, the narrow occlusal surface, the absence of mineralization and the upwards pointing rostrum with the lower jaw of the belemnite *Hibolithes* (Klug et al. 2010b) as well as with the lower belemnite jaw figured by Keupp and Mitta (2015: Fig. 24). Since PIMUZ 6077 is too large to fit into the body chamber of an adult *Leioceras* and the lower jaw of *Leioceras* has a different morphology (Mitta et al. 2018: Fig. 3), we suggest that this specimen represents the lower jaw of a belemnite.

In our opinion, the upper jaw PIMUZ 6076 (Fig. 2d) is a characteristic belemnite jaw. It shares the long and downward pointing rostrum of the upper jaw with that of *Hibolithes* (Klug et al. 2010b). The same long and spine-like rostrum combined with a rather long hood is present in the belemnite upper jaw illustrated by Keupp and Mitta (2015: Fig. 22). Like PIMUZ 6077, it does not resemble known jaws of Jurassic ammonoids and is much too large for *Leioceras*. Also, the rostrum of PIMUZ 6076 is significantly longer and more claw-like than in the jaws known from Mesozoic gladius-bearing octobrachians (Klug et al. 2005, 2010a; Nixon 2015). Thus, we conclude that PIMUZ 6076 is an upper jaw of a large belemnite.

Since reasonably preserved belemnite jaw remains are known only from *Hibolithes* (Klug et al. 2010b), early Albian *Neohibolithes* or ?*Conoteuthis* (Lehmann et al. 2016), Russian specimens described by Keupp and Mitta (2015: Figs. 22, 24) in open nomenclature, and the vague carbonaceous shadows from Toarcian belemnites (Reitner and Urlichs 1983), a taxonomic assignment of the Aalenian belemnite jaws to known belemnite taxa based on rostra must rely exclusively on size and proportions. For this purpose, we measured mantle lengths, rostrum lengths and jaw lengths of some Jurassic coleoids (Fig. 4, Table 1). In order to obtain an estimate of the corresponding rostrum length of both belemnite jaws, we also put rostrum lengths into relation with mantle and jaw lengths. Following this reasoning, we estimate the mantle length for the lower jaw PIMUZ 6077 to measure about 560 mm (using a ratio of 0.05, based on values from Toarcian coleoids, see Table 1) and for the upper jaw PIMUZ 6076, we calculated an estimated mantle length of 200 mm (using the ratio of 0.09 of *Hibolithes*). In any case, both jaws likely belonged to a large belemnite with rostra between 100 and 300 mm in length, assuming that the rostrum makes up about 50% of mantle length as in Toarcian belemnites. This suggests that they belonged to the largest species from the Opalinus Clay, namely *Acrocoelites conoideus*, because other belemnites known from this locality did not exceed 100 mm rostrum length.

**Table 1:** Measurements (in mm) and ratios of Jurassic coleoid bodies, gladii, rostra and jaws

Taxon	Age	Animal	Rostrum	Mantle/gladius/	Lower jaw	Upper jaw	Lower jaw/mantle	Upper jaw/mantle	Source	Remarks
*Acro. raui*	Toarc.	350	160	250	10		0.04		Reitner and Urlichs 1983	Deformed
*Pass. paxillosa*	Toarc.	344	110	240	9		0.038		Reitner and Urlichs 1983	Deformed
*Clark. conocauda*	Toarc.	240	64	150	9	9	0.06	0.06	Jenny et al. 2019	
*Geo. simplex*	Toarc.			200	20	20	0.1	0.1	Here	
*Hibolithes semisulcatus*	Kimm.	*450*	*150*	260	10	20	0.038	0.077	Klug et al. 2010b	No rostrum
*Tr. hastiformis*	Kimm.			268	28		0.104		Klug et al. 2005	No arms, SMNS 65344
*Tr. hastiformis*	Kimm.			255	18	24	0.071	0.094	Klug et al. 2005	No arms, SMNS 65345
*Plesio. prisca*	Kimm.			220		20		0.091	Klug et al. 2010b	
*Plesio. prisca*	Kimm.	*320*		*230*	21	20	0.0911	*0.087*	Klug et al. 2015	
*Lepto. gigas*	Tithon.	720		510	*28*	*30*	0.055	*0.059*	Klug et al. 2005	
Lower jaw, PIMUZ 6077	Aalen.		*280*	*560*	28		0.05		Here	
Upper jaw, PIMUZ 6076	Aalen.		*100*	*200*		18		*0.09*	Here	
*Acro. quenstedti*	Aalen.		95		*9.5*	*9.5*	*0.1*		Here	
*Acro. conoideus*	Aalen.		169		*16.9*	*16.9*	*0.1*		Here	

Values in italics indicate uncertain values, extrapolated values or values reconstructed using proportions from other taxa

*Acro Acrocoelites* (Belemnitida)*, Clark Clarkeiteuthis* (Diplobelida)*, Hib Hibolithes* (Belemnitida)*, Lept Leptoteuthis* (gladius-bearing Octobrachia)*, Geo Geopeltis* (gladius-bearing Octobrachia)*, Pass Passaloteuthis* (Belemnitida)*, Plesio Plesioteuthis* (gladius-bearing Octobrachia)*, Tr Trachyteuthis* (gladius-bearing Octobrachia)

**Fig. 5:**
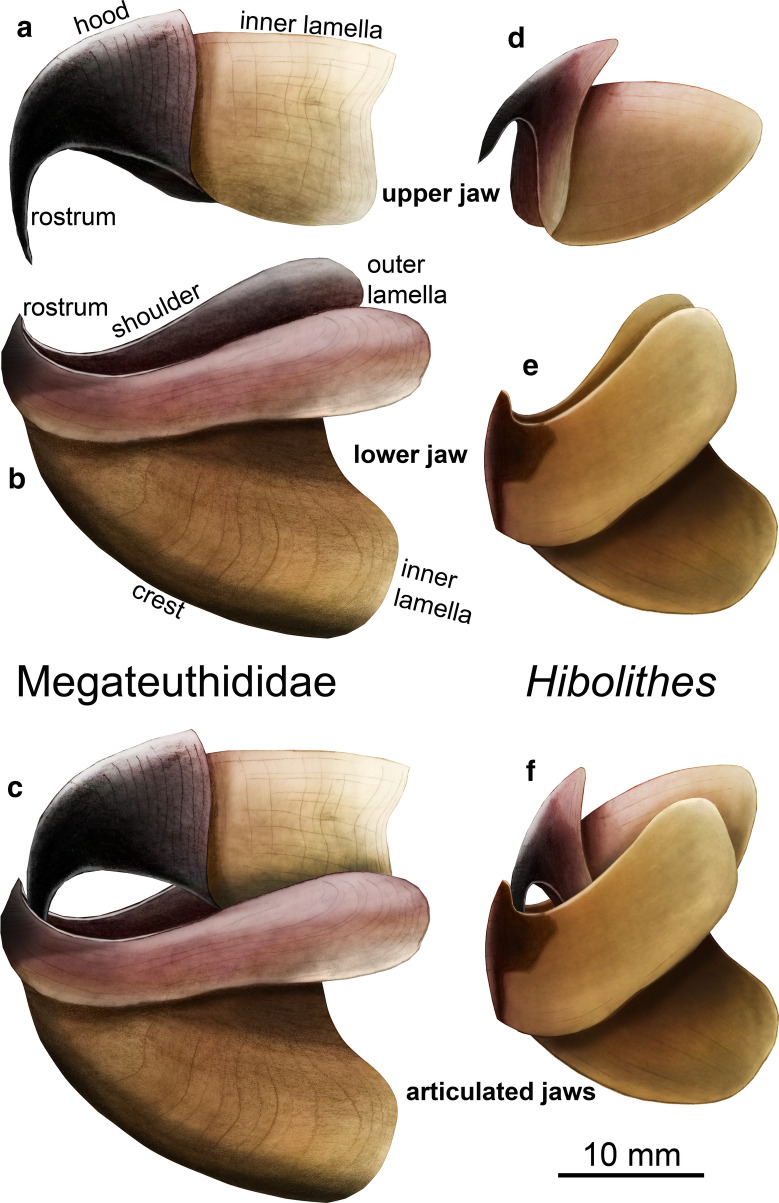
Reconstructions of Jurassic belemnite jaws. **a–c** *Acrocoelites conoideus* from the Swiss Aalenian. **d–f** *Hibolithes semisulcatus* from the German Kimmeridgian, modified after Klug et al. (2010b); the inner lamella of the lower jaw was enlarged

In contrast to the material published by Klug et al. (2010b), the new material permits a more accurate reconstruction of the complete belemnitid jaw (Fig. 5d–f). While in *Hibolithes*, the posterior parts of both jaws were hardly discernible, the Aalenian lower jaw described here (Fig. 5a–c) shows the outlines and proportions of inner and outer lamellae quite well. Accordingly, the inner lamella of *Hibolithes* was likely larger and longer (Fig. 5e) than suggested by Klug et al. (2010b). As far as the upper jaw is concerned, the extremely long and pointed, almost tooth-like rostrum of the megateuthidid *Acrocoelites* (Fig. 5a) appears to be characteristic for belemnites. It is conceivable that the pointed rostrum aided in capturing, holding and immobilizing slippery and agile prey as suggested for *Clarkeiteuthis* by Jenny et al. (2019).

## Conclusions

Here, we describe three cephalopod jaws from the Aalenian Opalinum Clay Formation of northern Switzerland. These jaws primarily lacked mineralized portions. Accordingly, such jaws are very rarely preserved and thus, we discuss their systematic affinities.

**Fig. 6:**
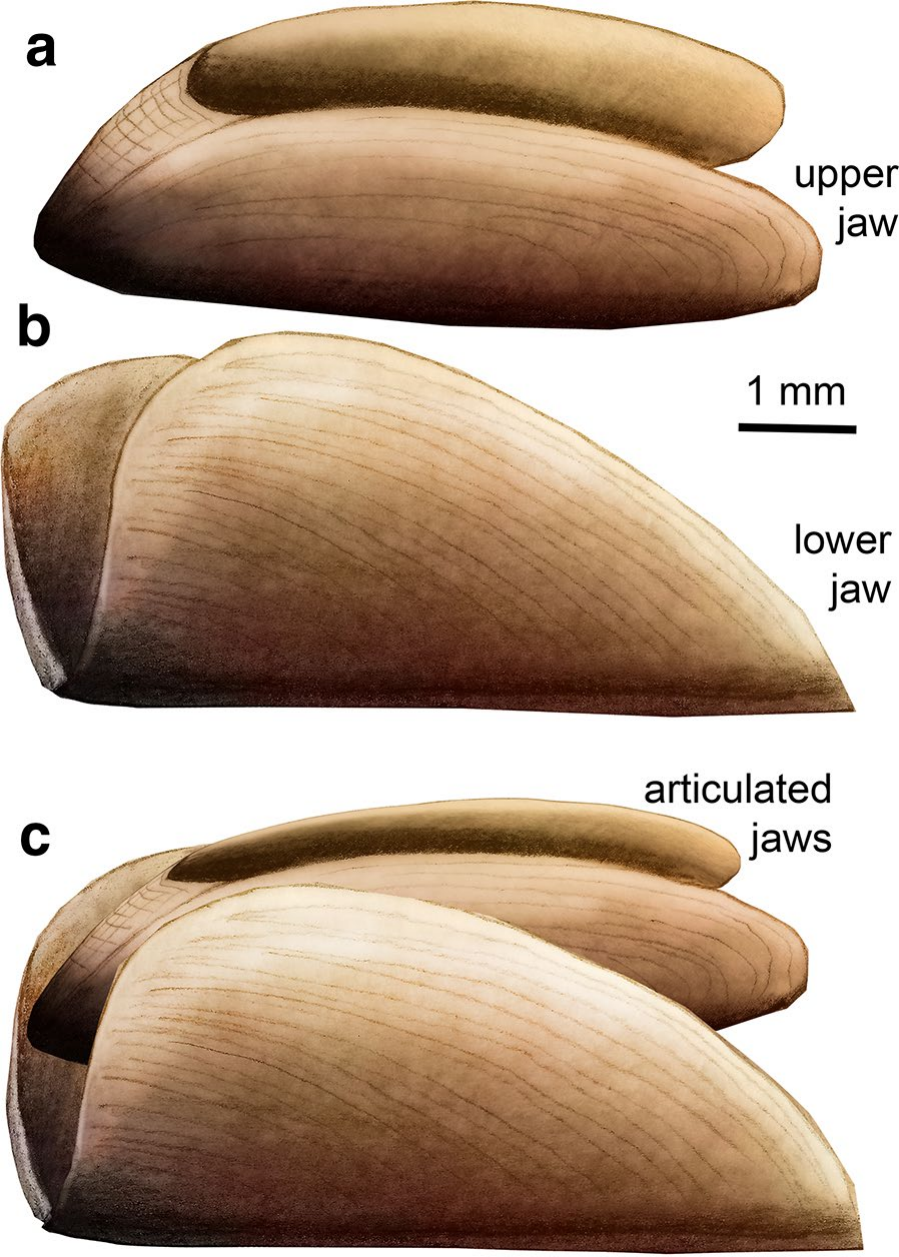
Reconstructions of the jaws of *Leioceras opalinum* (Reinecke 1818) from the Aalenian. **a** upper jaw after PIMUZ 5942. **b** lower jaw after the specimens shown in Mitta et al. (2015). **c** articulated jaws; the deep posterior incision allows movement in the body chamber where the preceding whorl restricts the whorl section

The smallest jaw shares several characters with the upper jaws of some Mesozoic ammonoids. Since *Leioceras opalinum* is by far the most common ammonite in the host strata and the elongate shape coincides with the elongate lower jaws of that ammonite, we assign it to this species with some reservation since it was not found within the conch of the according ammonite. This jaw is of interest because previously, only the lower jaws of *Leioceras* were known (Fig. 6).

The other two jaw elements share several morphological characters with that of the belemnite *Hibolithes* from the Late Jurassic of southern Germany. According to their dimensions, we suggest that these two jaw elements represent upper and lower jaws that belonged to the megateuthidid *Acrocoelites conoideus*, the largest belemnite of the Opalinum Clay Formation. Both jaws are compacted but rather complete. Their exceptional preservation helped to reconstruct the jaws of the largest belemnite family Megateuthididae for the first time and to improve the reconstruction of those of *Hibolithes*. The new material reflects how little is still known about the anatomy of Mesozoic coleoids and of belemnites in particular.

## Data Availability

All specimens illustrated and described are stored at the Paläontologische Institute und Museum of the Universität Zürich.
